# Supplementary material for: Characterization of mitochondrial health from human peripheral blood mononuclear cells to cerebral organoids derived from induced pluripotent stem cells
Source: Sci Rep. 2021 Feb 25;11:4523. doi: 10.1038/s41598-021-84071-6 (PMC7907388; doi:10.1038/s41598-021-84071-6)
Supplement: Supplementary file 1 — Supplementary Information. [file 41598_2021_84071_MOESM1_ESM.pdf]

Supplementary Information for

Characterization of mitochondrial health from human peripheral blood mononuclear cells to cerebral organoids derived from induced pluripotent stem cells

Angela Duong[1,2], Alesya Evstratova[1], Adam Sivitilli[3], J. Javier Hernandez[4,5], Jessica Gosio[4,5], Azizia Wahedi[6], Neal Sondheimer[4,6], Jeff L. Wrana[4,5], Jean-Martin Beaulieu[1]*, Liliana Attisano[3,6]*, Ana C. Andreazza[1,2,7]*.

[1]Department of Pharmacology and Toxicology, University of Toronto, Toronto, ON, M5S 1A8, Canada.
[2]Centre for Addiction and Mental Health, Toronto, ON, M5T 1R8, Canada.
[3]Department of Biochemistry, University of Toronto, Toronto, ON, M5S 1A8, Canada.
[4]Department of Molecular Genetics, University of Toronto, Toronto, ON, M5S 1A8, Canada.
[5]Lunenfeld-Tanenbaum Research Institute, Toronto, ON, M5G 1X5, Canada.
[6]Program in Genetics and Genome Biology, Peter Gilgan Centre for Research and Learning, The Hospital for Sick Children, Toronto, ON, M5G 0A4, Canada.
[7]Donnelly Centre for Cellular and Biomolecular Research, University of Toronto, Toronto, ON, M5S 3E1, Canada.
[8]Department of Psychiatry, University of Toronto, Toronto, ON, M5S 1A8, Canada.

*Correspondence to: martin.beaulieu@utoronto.ca (J.M.B.); liliana.attisano@utoronto.ca (L.A.); ana.andreazza@utoronto.ca (A.C.A.)

This PDF file includes:

Tables S1 to S11
Figures S1 to S2

Clinical and Demographic Characteristics	
Sample Identification	MS005-2014
Sample Type	Whole Blood
Informed consent	Yes
Age	33
Sex	Female
BMI	24.6
Medication	None reported
Ethnicity	Caucasian
BP	120/92

Table S1. Clinical and demographic characteristics of the donor. BMI, body mass index; BP, blood pressure.

	Antigen	Host	Dilution	Source	Identifier
iPSC	OCT4	Rabbit	1:400	Invitrogen	A24867
	SOX2	Rat	1:100	Invitrogen	A24759
	SSEA4	Mouse	1:100	Invitrogen	A24866
	TRA-1-60	Mouse	1:100	Invitrogen	A24868
CO	SOX2	Goat	3:200	R&D	AF2018
	NeuN	Rabbit	1:500	Cell Signaling	12943S
Mitochondria	TOMM-20	Rabbit	1:100	Abcam	ab186734
	NDUFS3	Mouse	1:100	Abcam	ab110246
	SDHA	Mouse	1:200	Abcam	ab14715
	UQCRC1	Mouse	1:50	Abcam	ab110246
	COXIV	Mouse	1:200	Abcam	ab33985
	ATP Synthase β	Mouse	1:500	Invitrogen	A-21351

Table S2. List of primary antibodies used for fluorescent immunohistochemistry experiments. OCT4, octamer-binding transcription factor 4; SOX-2, sex determining region Y-box 2; SSEA4, stage-specific embryonic antigen 4; TRA-1-60, podocalyxin; NeuN, neuronal nuclei; TOMM-20, translocase of outer mitochondrial membrane 20; NDUFS3, NADH:ubiquinone oxidoreductase core subunit S3; SDHA, succinate dehydrogenase complex flavoprotein subunit A; UQCRC1, ubiquinol-cytochrome C reductase core protein I; COXIV, cytochrome c oxidase subunit IV; ATP Synthase β, ATP synthase beta subunit; iPSC, induced pluripotent stem cells; CO, cerebral organoid.

	iPSC CO	H9 hESC CO
Electrophysiology	4	4
Cellular IF	2	2
Mitochondria	8	8
TOTAL	14	14

Table S3. Total number of whole cerebral organoids produced and allocated for each assessment. iPSC CO induced pluripotent stem cells-derived cerebral organoids; H9 hESC CO, H9 human embryonic stem cells- derived cerebral organoids; IF, immunofluorescence.

Assessment	Sample type	Specific Endpoints	Biological Replicates	Technical Replicates
Characterization	iPSC	Karyotype	2	10
		Alkaline phosphatase	3	3
		Pluripotency validation	3	3
	H9 hESC CO and iPSC CO	Cellular characterization	2	2
Neuronal Activity	H9 hESC CO and iPSC CO	Action potential	4	7-12
		Spontaneous activity	4	4-8
		Na+ and K+ currents	4	3-9
Mitochondrial Function	PBMC	MitoTracker	1	3
		MMP	1	3
		OXPHOS	1	3
		ATP	1	3
		mtDNA genetics	1	2
		TEM	1	10
	H9 hESCs and iPSCs	MitoTracker	3	3
		MMP	3	3
		OXPHOS	3	5
		ATP	3	2
		mtDNA genetics	3	1-3
		TEM	2	10
	H9 hESC CO and iPSC CO	MitoTracker	3	3
		MMP	4	3
		OXPHOS	3	5
		ATP	3	3
		mtDNA genetics	3	1-3
		TEM	1	10

Table S4. Summary of biological and technical replicates used for each experiment. PBMC, peripheral blood mononuclear cells; iPSC, induced pluripotent stem cells; H9 hESC, H9 human embryonic stem cells; CO, cerebral organoids; MMP, mitochondrial membrane potential; OXPHOS; oxidative phosphorylation; ATP, adenosine triphosphate; mtDNA, mitochondrial DNA; TEM, transmission electron microscopy; Na+, sodium; K+, potassium.

PCR Primers for Sex Determination		Size
SRY F	CATGAACGCATTCATCGTGTGGTC	254 bp
SRY R	CTGCGGGAAGCAAACTGCAATTCTT	
PARP F385	AAGCTTTCCAGGAGATCCTAAC	498 bp
PARP R882	CCTTCACTGTAGGTCCCAAAT	

Table S5. PCR primers used for sex determination as a quality control. SRY, sex determining region of Y-chromosome; PARP, poly(ADP-ribose) polymerase; F, forward; R, reverse; bp, base pair.

Sample	PARP	SRY	Sex
PBMC	+	-	F
iPSC	+	-	F
iPSC CO	+	-	F
**Controls**			
PC – SH-SY5Y	+	-	F
WG1669	+	+	M

Table S6. Sex determination as a quality control across all stages of cerebral organoid generation. There is preservation of the female karyotype across PBMC, iPSC and iPSC CO. SH-SY5Y human female neuroblastoma cell line was used as a female control and WG1669 male patient was used as a male control. PBMC, peripheral blood mononuclear cells; iPSC, induced pluripotent stem cells; H9 hESC, H9 human embryonic stem cells; CO, cerebral organoids; PC, positive control; SH-SY5Y, human neuroblastoma cell line; SRY, sex determining region of Y-chromosome; PARP, poly(ADP-ribose) polymerase; F, female; M, male.

Primers for mtDNA amplification for Illumina sequencing	
MT-COIII F	TCACAATTCTAATTCTACTGA
MT-COIII R	CGGATGAAGCAGATAGTGAGG
MT16425 F	CCGCACAAGAGTGCTACTCTCCTC
MT16426 R	GATATTGATTTCACGGAGGATGGTG

Table S7. Primers used for mtDNA amplification and sequencing. MT, mitochondrial; F, forward; R, reverse.

	PBMC	iPSC	iPSC-derived CO	NC - SH SY5Y
A73G	+	+	+	-
A153G	+	+	+	-
T195C	+	+	+	-
G225A	+	+	+	-
A263G	+	+	+	+
A750G	+	+	+	+
G769A	+	+	+	-
A1438G	+	+	+	+
G1719A	+	+	+	-
A2706G	+	+	+	-
A4769G	+	+	+	+
T6221C	+	+	+	-
C6371T	+	+	+	-
C7028T	+	+	+	-
A8860G	+	+	+	+
T9722C	+	+	+	-
G11719A	+	+	+	-
C12705T	+	+	+	-
G13368A	+	+	+	-
A13966G	+	+	+	-
T14470C	+	+	+	-
C14766T	+	+	+	-
A15326G	+	+	+	+
G15650A	+	+	+	-
A15924G	+	+	+	-
T16189C	+	+	+	-
C16223T	+	+	+	-
C16278T	+	+	+	-
T16519C	+	+	+	+

Table S8. Mitochondrial DNA (mtDNA) variants for the X2g haplogroup across PBMCs, iPSCs and iPSC-derived COs validated by whole mtDNA sequencing and short mtDNA fragment sequencing. SH-SY5Y human neuroblastoma cell line was used as a negative control (NC), validated by only short fragment mtDNA sequencing. (+) denotes positive or presence; (-) denotes negative or absence. PBMC, peripheral blood mononuclear cells; iPSC, induced pluripotent stem cells; CO, cerebral organoids.

PCR Primers		Sequencing primers		SNPs sequenced				
F13464	CAGCCTAGCATTAGCAGGAATA	F14368	CAATCCTACCTCCATCGCTAAC	T14470C	C14766T			
R15211	GTATGGGATGGCGGATAGTAAG							
F458	CCTCCCACTCCCATACTACTAA	R1927	CTTAGGTAGCTCGTCTGGTTTC	A1438G	G1719A			
R1997	GGTTTGTCGCCTCTACCTATAAA							
F4708	TCTCCGGACAATGAACCATAAC	R5216	GAGGAGGGTGGATGGAATTAAG	A4769G	C5111T			
R6882	GTGTGGCGAGTCAGCTAAATA	F5993	CACAGCTCTAAGCCTCCTTATT	T6221C	C6371T			
F14562	CCACACCGCTAACAATCAATAC	F15264	CCACCCTCACACGATTCTTTAC	A15326G	G15650A	A15924G		
R16511	GTAGGAACCAGATGTCGGATAC							
F8474	CCCTCACCAAAGCCCATAAA	F8804	CACCAACCACCCAACTATCT	A8860G				
R11031	CGTGATAGTGGTTCACTGGATAAG	F9318	CACTCCATAACGCTCCTCATAC	T9722C				
F11644	CCTCGTAGTAACAGCCATTCTC	R11775	CTGTGAGTGCGTTCGTAGTT	G11719A				
R14130	GGTTAGGATGAGTGGGAAGAAG	F13595	GCGCCTATAGCACTCGAATAA	A13966G				
		R13451	GAGGTTGAAGTGAGAGGTATGG	C12705T	G13368A			
F15323	GCAACACTCCACCTCCTATTC	F458	CCTCCCACTCCCATACTACTAA	A750G	G769A			
R943	CTCTTTACGCCGGCTTCTATT	R16548	GGGAACGTGTGGGCTATTTA	T16189C	C16223T	C16278T		
		F16536	CCCACACGTTCCCCTTAAATAA	A73G	A153G	T195C	G225A	A263G
		R34	CGTGAGTGGTTAATAGGGTGATAGAC	T16519C				
F5317	CCACCATCACCCTCCTT	F6357	CTAGCAGGTGTCTCCTCTATCT	C7028T				
R7608	CCTACTTGCGCTGCATGTGCC							
F1562	GTAACATGGTAAGTGTACTG	F2209	GCTCAACACCCACTACCTAAA	A2706G				
R3717	GGCTACTGCTCGCAGTG							

Table S9. PCR primers and sequencing primers used for the determination of the mitochondrial DNA haplogroup, X2g. F, forward; R, reverse; SNPs, single nucleotide polymorphisms.

PCR Primers for mtDNA Copy Number		
mtDNA	MT-ND1 F	ATGGCCAACCTCCTACTCCT
	MT-ND1 R	CTACAACGTTGGGGCCTTT
Nuclear DNA	ß2M F	TGCTGTCTCCATGTTTGATGTATCT
	ß2M R	TCTCTGCTCCCCACCTCTAAGT

Table S10. PCR primers used for mtDNA copy number determination. MT-ND1, mitochondrially encoded NADH:ubiquinone oxidoreductase core subunit 1; ß2M, Beta 2 Microglobulin; F, forward; R, reverse.

	Resting membrane potential (mV)			Membrane capacitance (pF)			Membrane resistance (MOhm)		
	Type 1	Type 2	Type 3	Type 1	Type 2	Type 3	Type 1	Type 2	Type 3
iPSC CO	42 ± 2	42 ± 3	47 ± 3	20 ± 3	29 ± 3	39 ± 3	1137 ± 214	847 ± 114	558 ± 48
H9 hESC CO	41 ± 4	47 ± 2	56 ± 3	22 ± 3	29 ± 3	32 ± 2	834 ± 175	577 ± 74	574 ± 102

Table S11. Electrophysiological properties of neurons from iPSC-derived CO and H9 hESC-derived CO. Type 1, immature neurons; type 2, developing neurons; type 3, mature neurons; iPSC, induced pluripotent stem cells; H9 hESC, H9 human embryonic stem cells; CO, cerebral organoids.

A

B

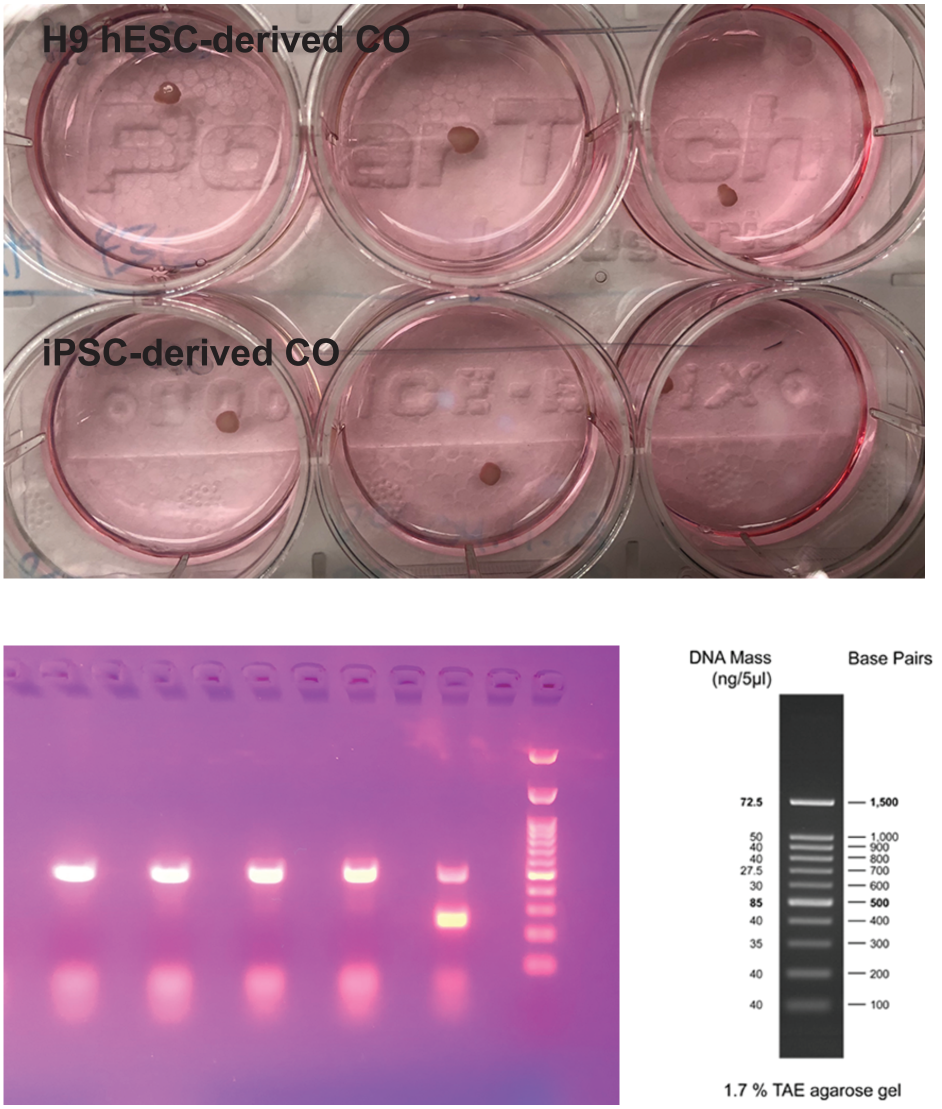

Fig. S1. Sex determination as a quality control. (A) Representative cerebral organoid culture in a 6-well plate, H9 hESC-derived CO (top row) and iPSC-derived CO (bottom row). **(B)** A representative gel image of SRY and PARP PCR products. Left to right: PBMC, iPSC, iPSC-derived CO, SH-SY5Y neuroblastoma cell line, a male control and 100bp ladder.

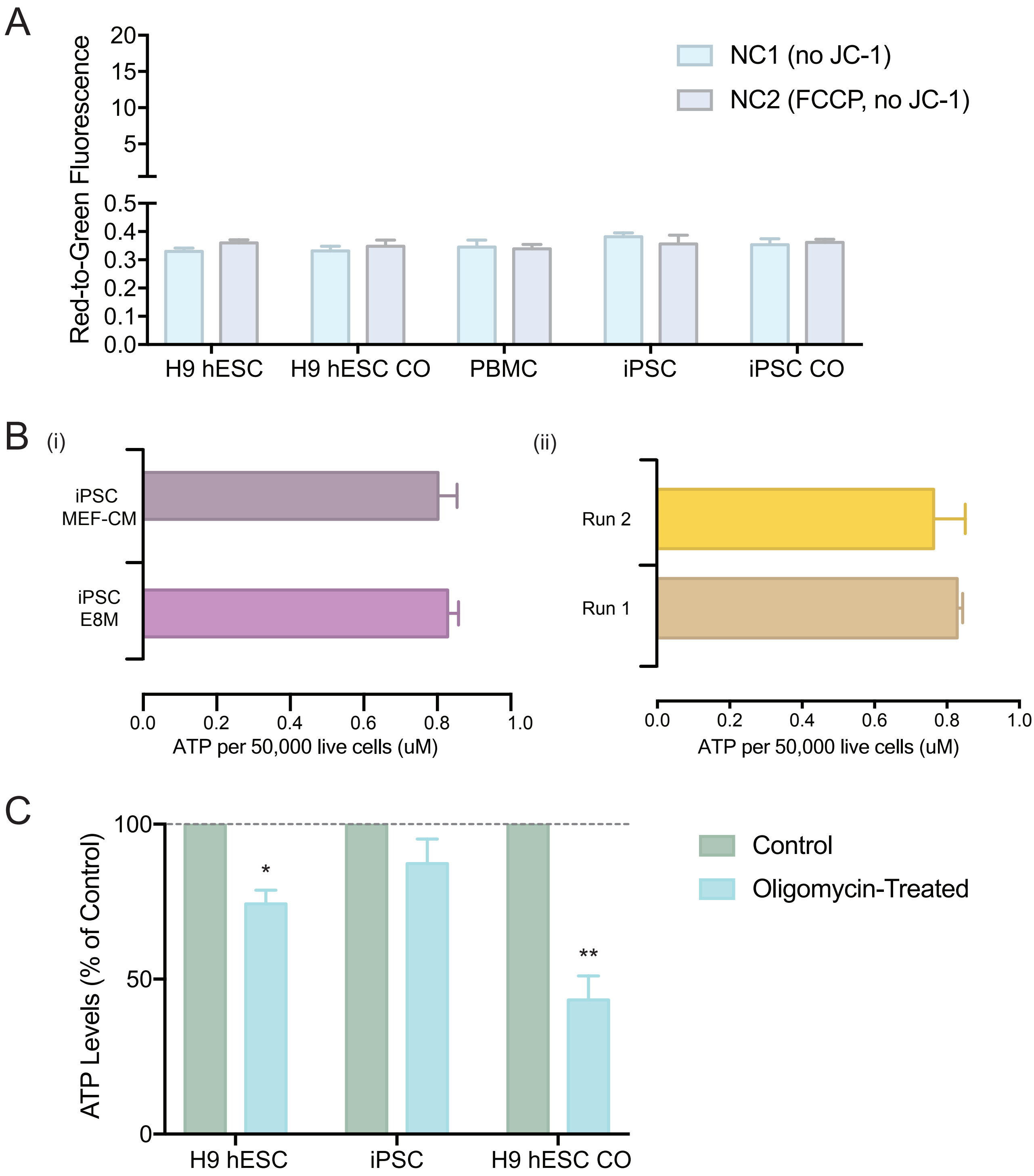

Fig. S2. Mitochondrial function experimental quality control and proof-of-concept. (A) Bar graph summarizing mitochondrial membrane potential as red-to-green fluorescence ratio across CO generation in samples treated without JC-1 (negative control 1, NC-1) and those treated with FCCP and without JC-1 (negative control 2, NC-2), bars, mean ± SD. **(B)** Bar graph showing intracellular ATP levels in iPSCs cultured in Mouse Embryonic Fibroblast (MEF)-Conditioned Media and Essential 8 Media. bars, mean ± SD (i). Bar graph showing the intracellular ATP levels in iPSCs in two independent experimental runs, bars, mean ± SD (ii). **(C)** Bar graph showing ATP levels expressed as percent of control in H9 hESCs, iPSCs and H9 hESC COs treated with oligomycin, bars, mean ± SD. *$p<0.01$; **$p<0.001$. *P*-values were determined using the independent samples *t*-test.
